# A Truncated Endogenous U6 Promoter Enables High-Efficiency CRISPR Editing in Flax (*Linum usitatissimum* L.)

**DOI:** 10.3390/plants14071142

**Published:** 2025-04-06

**Authors:** Feifei Li, Min Xue, Dongliang Guo, Leilei Zhu, Yuandong Li, Liqiong Xie

**Affiliations:** 1 Xinjiang Key Laboratory of Biological Resources and Genetic Engineering, College of Life Science and Technology, Xinjiang University, Urumqi 830046, China; lifeifei@stu.xju.edu.cn (F.L.); 13399933904@163.com (M.X.); gdl520jhx@163.com (D.G.); zhuleilei@stu.xju.edu.cn (L.Z.); liyuandong@stu.xju.edu.cn (Y.L.); 2 College of Smart Agriculture, Xinjiang University, Urumqi 830046, China

**Keywords:** flax (*Linum usitatissimum* L.), U6 promoter, activity, truncation, reporter gene, CRISPR/Cas9

## Abstract

Functional U6 promoters are widely utilized in CRISPR gene editing systems for crops. The identification of endogenous U6 promoter activity and the establishment of CRISPR/Cas9 gene editing systems in various crops can enhance the efficiency and accuracy of gene editing in molecular breeding. In this study, four U6 snRNAs were identified in the genome of the oil flax (*Linum usitatissimum* L.) cultivar Longya 10, which exhibit high homology with the promoter regions of Arabidopsis thaliana U6 snRNA. We cloned and constructed fusion expression vectors with U6 promoter-driven dual-luciferase reporter genes. Transient transformation of flax and *Nicotiana benthamiana* was performed to measure the relative activity of dual luciferase. The *U6-4* on chromosome 14 showed the highest transcriptional activity. Truncations of varying lengths from the 5′ end of this promoter were tested, revealing that a 342 bp U6 promoter fragment possesses high transcriptional activity and an optimal length. Subsequently, we constructed a CRISPR/Cas9 gene editing vector with *LuU6-5P/AtU6-P* driving *LusPDS* sgRNA. Agrobacterium-mediated infection of flax hypocotyls yielded transgenic albino flax shoots. DNA from these shoots was used as a template to amplify *LusPDS* fragments, which were then sequenced. Sequencing analysis revealed that CRISPR/Cas9 vectors using *Lu14U6-4-5P* achieved higher editing frequencies at *LusPDS* compared to *AtU6-P*-driven systems.

## 1. Introduction

Flax (*Linum usitatissimum* L.) is globally recognized for its abundant fiber and nutrient-rich seed oil, making it a vital economic crop [1,2]. Flaxseed oil is an excellent source of alpha-linolenic acid (ALA) and omega-3 fatty acids, positioning it as a valuable ingredient in specialty foods, animal feed, and industrial applications [3,4]. Furthermore, flax stem fibers are extensively employed in the production of high-quality textiles, geo-textiles, composite materials, and pulp for the paper industry. Due to its versatility and economic significance, flax is often referred to as the “Queen of Fibers”. The increasing demand for flax-derived products highlights the urgent need to develop high-yielding, stress-tolerant varieties through advanced biotechnological methods, such as genetic transformation and genome editing, which have become pivotal objectives in contemporary flax breeding programs.

The Clustered Regularly Interspaced Short Palindromic Repeats (CRISPR)/CRISPR-associated protein 9 (Cas9) system has revolutionized functional genomics and crop improvement due to its simplicity, high efficiency, cost-effectiveness, and remarkable versatility [5]. Unlike conventional knockout/knock-in transgenic techniques used in molecular breeding, CRISPR/Cas9 enables precise modifications—including deletions, insertions, and site-specific mutations—at targeted genomic regions, thus facilitating the introduction of novel traits in plants [6]. To date, this technology has been applied to enhance over 25 plant species across more than 40 countries [7]. However, editing efficiency in flax remains constrained by species-specific factors, including promoter compatibility, expression levels of Cas9 and sgRNA, structural stability of sgRNA, and the design of the 20-nt spacer sequence [8,9,10,11]. Addressing these limitations is critical to unlocking the full potential of CRISPR/Cas9 in flax genome engineering.

In plant CRISPR/Cas9-based genome editing systems, the U6 and U3 promoters serve as critical regulatory elements for driving the transcription of sgRNA [12,13]. The U6 promoter is distinguished by its high transcriptional activity and a precise transcription start site (TSS) that begins with a guanine (G) nucleotide, which ensures accurate sgRNA synthesis. This accuracy reduces off-target effects by enhancing the pairing between sgRNA and its target sequence [14,15]. U6 and U3 promoters derived from model plants such as *Arabidopsis thaliana* and rice (*Oryza sativa*) have provided valuable references for gene editing systems across diverse plant species. However, heterologous expression studies indicate significant species-specific variations in U6 promoter activity. In certain plants, endogenous U6 promoters demonstrate superior performance compared to the widely used AtU6 promoter, especially when applied to recipient materials [9,16]. Furthermore, transcriptional activity can vary among different U6 promoters within the same species [17,18]. For example, in banana, the activity of the *MaU6c* promoter is approximately fourfold higher than that of the *OsU6a* promoter [19]. Although U6 promoters from rice, wheat (*Triticum aestivum*), and *Arabidopsis* have been successfully employed in specific species [20,21,22], identifying endogenous flax U6 promoters with high transcriptional activity remains essential for minimizing off-target effects and enhancing editing efficiency in phylogenetically distant species.

The core regulatory elements of the U6 promoter include the upstream sequence element (USE) at the −60 position, which modulates transcriptional activity, and the TATA-like box at −30, crucial for RNA polymerase III recognition and transcription initiation. As a type III promoter, the U6 promoter requires only these upstream elements and 4–5 consecutive thymine (T) residues at the 3′ end to function [23]. Studies in soybean (*Glycine max*), cotton (*Gossypium hirsutum*), and castor (*Ricinus communis*) indicate that endogenous U6 promoters achieve maximal activity at lengths of 350 bp, 300 bp, and 300 bp, respectively [24,25,26]. In gene editing vectors, compact promoter sequences are preferred to accommodate multiple target fragments and minimize restriction enzyme site interference. Thus, identifying the optimal length of flax U6 promoters with high transcriptional activity is a key focus for enhancing CRISPR/Cas9 system performance in flax genome engineering.

Due to its widespread cultivation in Eastern Europe, Central Asia, and North America, flax has become a cornerstone of economic development in northwestern China, where it drives poverty reduction and income growth for local farmers [27]. Leveraging the advantages of CRISPR/Cas9 technology in molecular breeding and crop improvement, this study emphasizes the importance of sgRNA transcription driven by U6 promoters and the necessity of identifying optimal endogenous U6 promoters in flax. In the oil flax cultivar Longya 10, four U6 snRNAs were identified in the genome. Using a dual-luciferase reporter system [28], transient transformation assays in flax and *Nicotiana benthamiana* revealed a highly transcriptionally active promoter on chromosome 14. Truncations of varying lengths from the 5′ end of this promoter were tested, and a 342 bp fragment (designated *LuU6-5P*) demonstrated the highest transcriptional activity. To compare editing efficiencies, CRISPR/Cas9 vectors expressing *Phytoene desaturase* (*LusPDS*) sgRNA driven by either *LuU6-5P* or *AtU6-P* were constructed. Agrobacterium-mediated transformation of flax hypocotyls yielded transgenic albino shoots. DNA from these shoots was used to amplify *LusPDS* fragments, which were sequenced to assess editing outcomes. The results showed that the *LuU6-5P*-driven vector successfully targeted *LusPDS*, achieving a 0.52% higher editing frequency compared to the *AtU6-P*-driven system.

## 2. Results

### 2.1. Sequence Analysis of Flax U6 Promoters: Structural Conservation and Functional Implications

U6 promoters, essential for RNA polymerase III-dependent transcription of sgRNAs in CRISPR/Cas9 systems, are characterized by conserved upstream sequence elements (USE) and TATA-like boxes critical for transcriptional initiation and fidelity [29]. These promoters exhibit species-specific variations in activity, necessitating the identification of endogenous sequences for optimized genome editing [30]. In this study, a BLAST (https://blast.ncbi.nlm.nih.gov/Blast.cgi) (accessed on 3 April 2025) search using a 120 bp conserved U6 snRNA sequence from *Arabidopsis thaliana* against the flax reference genome identified 11 candidate flax U6 snRNAs. Chromosomal distribution analysis revealed a predominant localization on chromosomes 13 and 14 (Table 1). After stringent screening, four U6 snRNA sequences were selected, all 106 bp in length and highly conserved, with a guanine (G) as the transcription start site (TSS).

**Figure 1:**
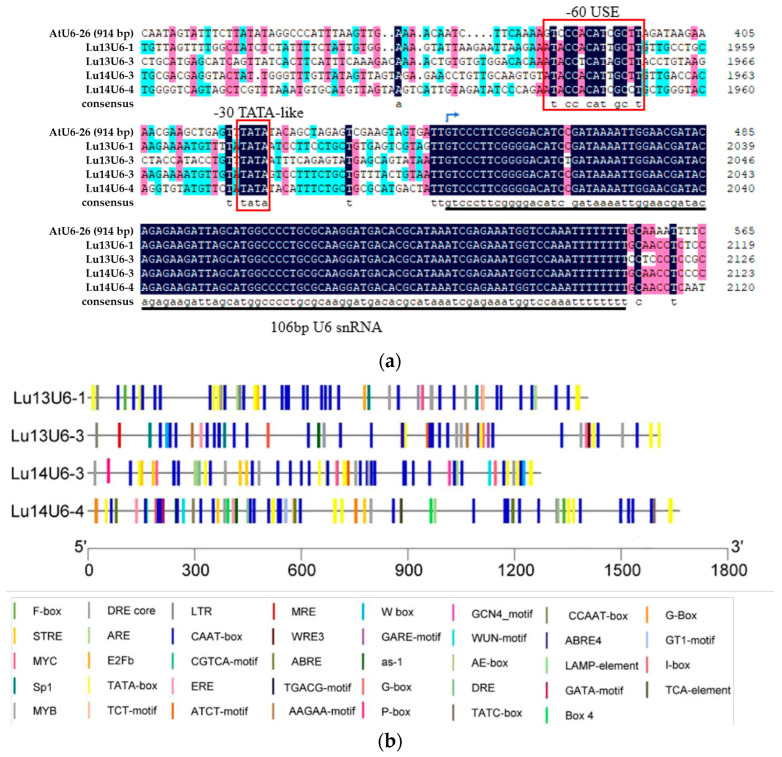
Analysis of the flax U6 promoter sequence. (**a**) Multiple sequence alignment of flax U6 snRNA genes and the *Arabidopsis AtU6-26* snRNA gene. Highly conserved regions are shaded in black. Key regulatory elements—the upstream sequence element (USE), TATA box, and U6 small nuclear RNA (snRNA) sequence—are demarcated with red boxes and underlining. The transcription start site (TSS) is indicated by a small arrow; (**b**) Cis-acting element analysis of the flax U6 snRNA promoter. Predicted regulatory motifs, including CAAT boxes and TATA-like elements, are annotated to illustrate their spatial distribution relative to the TSS.

The candidate flax U6 promoters were defined as 27 bp at the 5′ end of each U6 snRNA plus 2000 bp upstream of the TSS, predicted using online tools. These promoters, designated *Lu13U6-1*, *Lu13U6-3*, *Lu14U6-3*, and *Lu14U6-4*, were aligned with the *Arabidopsis AtU6-26* promoter. This alignment revealed conserved regulatory elements: a 30 bp TATA box and a 60 bp USE upstream of the TSS (Figure 1a), which are essential for RNA polymerase III binding and transcriptional activation. The spacing and sequence between these cis-acting elements were remarkably conserved. Cis-element analysis identified multiple transcription enhancers (e.g., CAAT boxes) and core elements (TATA boxes) across the four promoters (Figure 1b). Transcriptional activity correlated with promoter length, as longer sequences harbored more CAAT and TATA boxes. Visualization using TBtools further confirmed that the predicted TATA box proximal to the TSS aligned precisely with the position in *AtU6-26*, underscoring structural conservation (Figure 1a). Given the structural similarity and conserved elements shared between the four flax U6 promoters and the canonical *Arabidopsis thaliana* U6 promoter, their transcriptional activities could not be conclusively determined through preliminary analyses. Therefore, we conducted a systematic comparative analysis of transcriptional efficiency to identify optimal promoter candidates for CRISPR-based genome editing applications.

### 2.2. Transcriptional Validation of Endogenous Flax U6 Promoter Candidates

**Figure 2:**
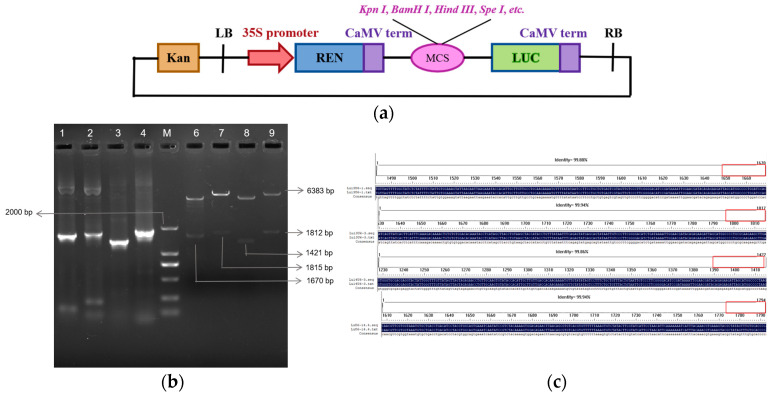
Cloning and double digestion verification of four candidate flax U6 promoters in expression vector construction. (**a**) Schematic diagram of the pGreenII0800-LUC vector; (**b**) construction of pGreenII0800 expression vectors for the four U6 promoters. Lanes: M (DL2000 DNA marker); Lane 1: *Lu13U6-1* (1670 bp); Lane 2: *Lu13U6-3* (1815 bp); Lane 3: *Lu14U6-3* (1421 bp); Lane 4: *Lu14U6-4* (1811 bp); Lanes 6–9: Corresponding double digestion verification of the constructed vectors; (**c**) sequencing verification of the pGreenII0800-LUC plant fusion expression vectors constructed with *Lu13U6-1*, *Lu13U6-3*, *Lu14U6-3*, and *Lu14U6-4*.

Promoter activity validation in planta is critical for optimizing CRISPR/Cas9 systems, as endogenous regulatory elements often outperform heterologous counterparts in driving sgRNA transcription [31,32,33]. This principle guided our evaluation of four candidate flax U6 promoters identified through genomic screening. Using four pairs of specific primers (Table A1) and genomic DNA from the oil flax cultivar Longya 10, PCR amplification successfully generated *Lu13U6-1* (1670 bp), *Lu13U6-3* (1815 bp), *Lu14U6-3* (1421 bp), and *Lu14U6-4* (1811 bp) promoter fragments, each containing restriction enzyme sites (Figure 2a). These fragments were ligated into the T5 cloning vector, transformed into T1 competent cells, and verified by double digestion and sequencing. Correctly sequenced fragments were subsequently cloned into the pGreenII0800-LUC expression vector using T4 ligase, followed by transformation into *E. coli* DH5α and validation via double digestion (Figure 2b,c). Recombinant plasmids were introduced into *Agrobacterium tumefaciens* GV3101 competent cells using a freeze–thaw method.

**Figure 3:**
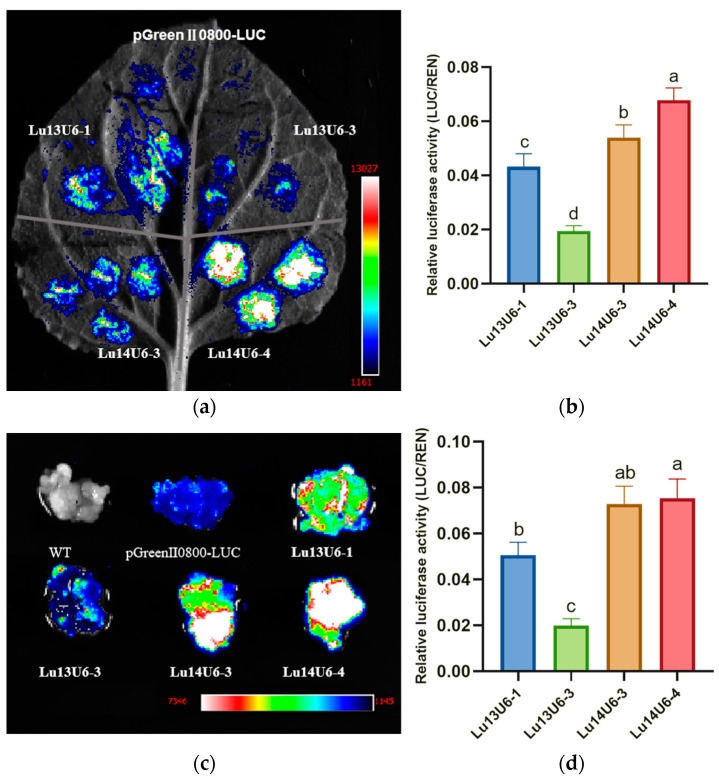
Dual-luciferase assay validation of transcriptional activity for four flax U6 promoters. (**a**) Luminescence imaging of dual-luciferase activity in transiently transformed *Nicotiana benthamiana* leaves; (**b**) LUC/REN enzymatic activity quantification for the four candidate flax U6 promoters in transiently transformed *N. benthamiana* leaves; (**c**) luminescence imaging of dual-luciferase activity in transiently transformed flax embryogenic callus tissues; (**d**) LUC/REN enzymatic activity quantification for the four candidate flax U6 promoters in transiently transformed flax callus tissues. (**b**,**d**) Values are means ± SD from at least four independent experiments; statistical significance was determined by analysis of variance (ANOVA) followed by Duncan’s test, with significant differences indicated by different lowercase letters (*p* < 0.05).

To evaluate transcriptional activity, Agrobacterium-mediated transient transformation was performed in *Nicotiana benthamiana* leaves and flax embryogenic callus tissues. Uninfected callus and the pGreenII0800-LUC empty vector served as negative controls. Luciferase activity in callus tissues was quantified 4 days post-transformation. In vivo imaging revealed luminescence in all transiently transformed tissues except wild-type controls (Figure 3a,c), confirming functional promoter activity. Statistical analysis of the tobacco leaf fluorescence signal shows that, compared with other candidate promoters, *Lu14U6-4* has much higher transcriptional activity. However, in flax callus, the transcriptional activities of *Lu14U6-3* and *Lu14U6-4* are similar (Figure 3b,d). Previous analysis of the cis-acting elements in these promoters indicates that *Lu14U6-4* contains more CAAT boxes (common cis-acting elements in promoter and enhancer regions) than *Lu14U6-3* (Figure 1b). Studies on endogenous promoters in apples suggest that the number of enhancer elements may affect transcriptional activity [34]. We speculate that a similar situation exists in flax.

Based on the principle that *Lu14U6-4* has more enhancer elements (such as CAAT boxes) than *Lu14U6-3* and its overall superiority in fluorescence signals, we chose *Lu14U6-4* for truncation experiments to further optimize the structure of endogenous promoters in the subsequent CRISPR/Cas9 editing system in flax.

### 2.3. Construction and Activity Validation of Truncated Flax LuU6 Promoter Expression Vectors

**Figure 4:**
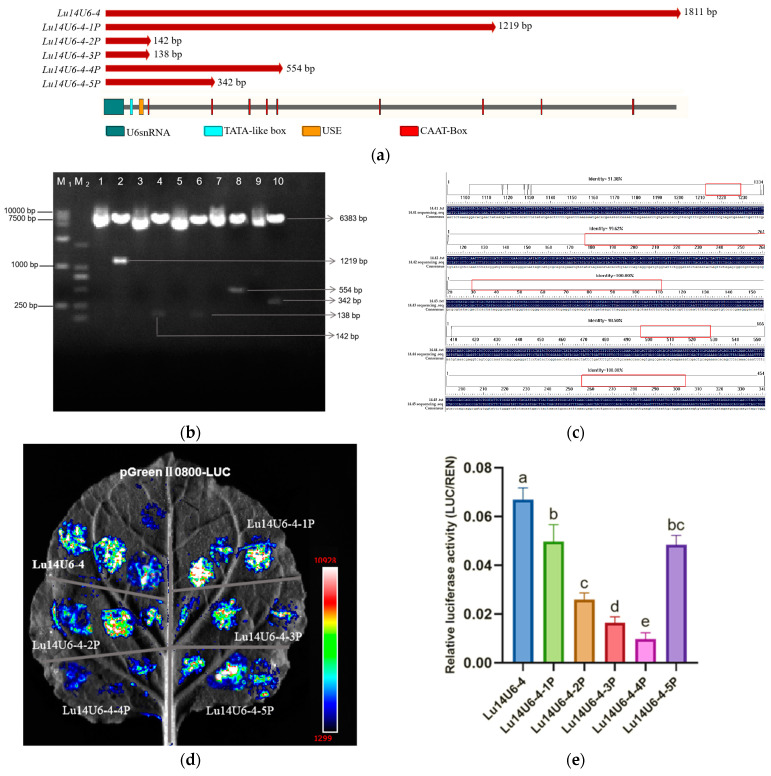

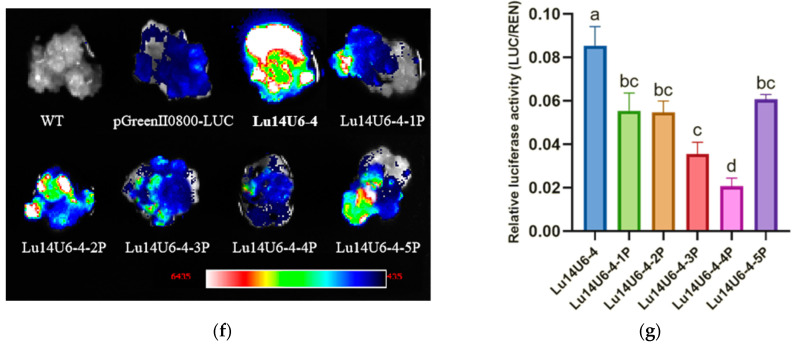
Construction and transient expression analysis of truncated flax U6 promoter vectors. (**a**) Schematic representation of 5′-end truncations of the flax U6 promoter; (**b**) construction of pGreenII0800 expression vectors for the truncated U6 promoters. Lanes: M1 (DL15000 DNA marker), M2 (DL2000 DNA marker); Lanes 1, 3, 5, 7, 9: Constructed pGreenII0800 vectors for *Lu14U6-4-1P* to *Lu14U6-4-5P*; Lanes 2, 4, 6, 8, 10: Corresponding double digestion verification of plasmids; (**c**) sequencing verification of the pGreenII0800-LUC expression vectors for the five truncated U6 promoters (*Lu14U6-4-1P* to *Lu14U6-4-5P*); (**d**) luminescence imaging of dual-luciferase activity in transiently transformed *Nicotiana benthamiana* leaves; (**e**) LUC/REN enzymatic activity quantification for the four candidate truncated U6 promoters in *N. benthamiana* leaves; (**f**) luminescence imaging of dual-luciferase activity in transiently transformed flax embryogenic callus tissues; (**g**) LUC/REN enzymatic activity quantification for the four candidate truncated U6 promoters in flax callus tissues. (**e**,**g**) Values are means ± SD from at least four independent experiments; statistical significance was determined by analysis of variance (ANOVA) followed by Duncan’s test, with significant differences indicated by different lowercase letters (*p* < 0.05).

In plants, cis-acting elements such as CAAT-box and GC motifs are critical for promoter activity. The CAAT-box, containing the core sequence 5′-CCAAT-3′/5′-CAAAT-3′, serves as a key regulatory element capable of binding diverse transcription factors. Based on predicted CAAT-box positions, the flax U6 promoter (*Lu14U6-4*) on chromosome 14 was truncated from the 5′ end to generate fragments of 1219 bp, 142 bp, 138 bp, 554 bp, and 342 bp (Figure 4a–c). These truncated promoters were designated *Lu14U6-4-1P*, *Lu14U6-4-2P*, *Lu14U6-4-3P*, *Lu14U6-4-4P*, and *Lu14U6-4-5P*. Following PCR amplification and verification by double digestion and sequencing, the fragments were ligated into the pGreenII0800-LUC expression vector. Correct ligation was confirmed through restriction enzyme digestion and sequencing.

Transient transformation assays using Agrobacterium-mediated delivery in *Nicotiana benthamiana* leaves and flax embryogenic callus revealed luminescence in all transformed tissues except wild-type controls (Figure 4d,f), confirming transcriptional activity of the truncated promoters. Statistical analysis demonstrated that the full-length *Lu14U6-4* exhibited the highest activity. Among the truncated variants, *Lu14U6-4-1P* (1219 bp) and *Lu14U6-4-5P* (342 bp) showed robust activity in tobacco leaves, while *Lu14U6-4-1P*, *Lu14U6-4-2P* (142 bp), and *Lu14U6-4-5P* displayed high activity in flax callus tissues (Figure 4e,g).

The application of CRISPR/Cas9 in plants may be hindered by low transformation efficiency and oversized vectors, particularly due to large components such as the Cas9 gene and the CaMV 35S promoter [35]. To address this, we prioritized compact endogenous U6 promoters for optimized vector design. Based on transcriptional activity in both flax callus and tobacco leaves, the 342 bp *Lu14U6-4-5P* promoter was selected for subsequent CRISPR/Cas9 editing experiments. This truncated promoter retained high transcriptional efficiency while minimizing vector size, aligning with strategies to enhance multiplex editing capabilities in flax. These findings emphasize the importance of balancing promoter length, cis-regulatory element composition, and vector design flexibility to advance precision genome editing in crops. The integration of endogenous promoters like *Lu14U6-4-5P* provides a practical framework for improving CRISPR/Cas9 efficiency in flax and related species.

### 2.4. Development and Genetic Transformation of CRISPR/Cas9 Gene Editing Vectors Driven by Endogenous U6 Promoters in Flax

**Figure 5:**
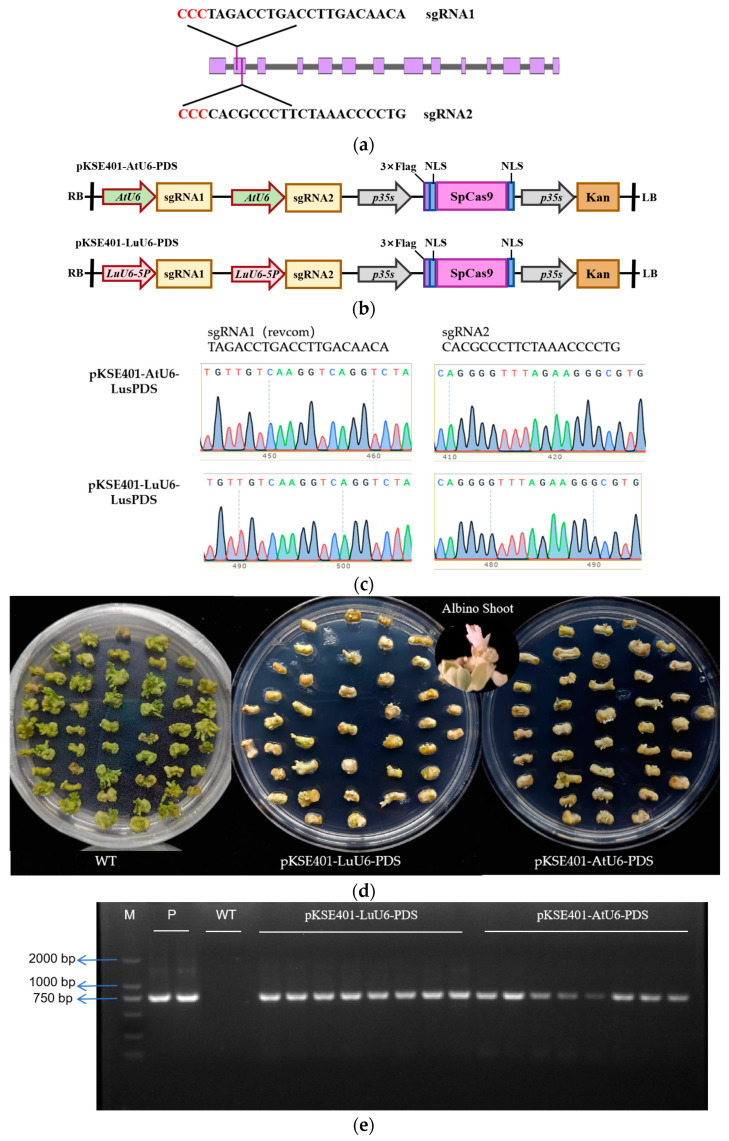
Construction and transformation of flax pKSE401-LuU6-PDS and pKSE401-AtU6-PDS editing vectors. (**a**) Selection of sgRNA target sites for *LusPDS*; (**b**) schematic diagrams of the pKSE401-LuU6-PDS and pKSE401-AtU6-PDS vectors; (**c**) sequencing verification of sgRNA regions in pKSE401-LuU6-PDS and pKSE401-AtU6-PDS vectors; (**d**) albino shoots generated from hypocotyls transformed with pKSE401-LuU6-PDS and pKSE401-AtU6-PDS vectors via Agrobacterium-mediated transformation; (**e**) PCR validation of exogenous T-DNA insertion in albino shoots using Cas9-specific primers.

**Figure A1:**
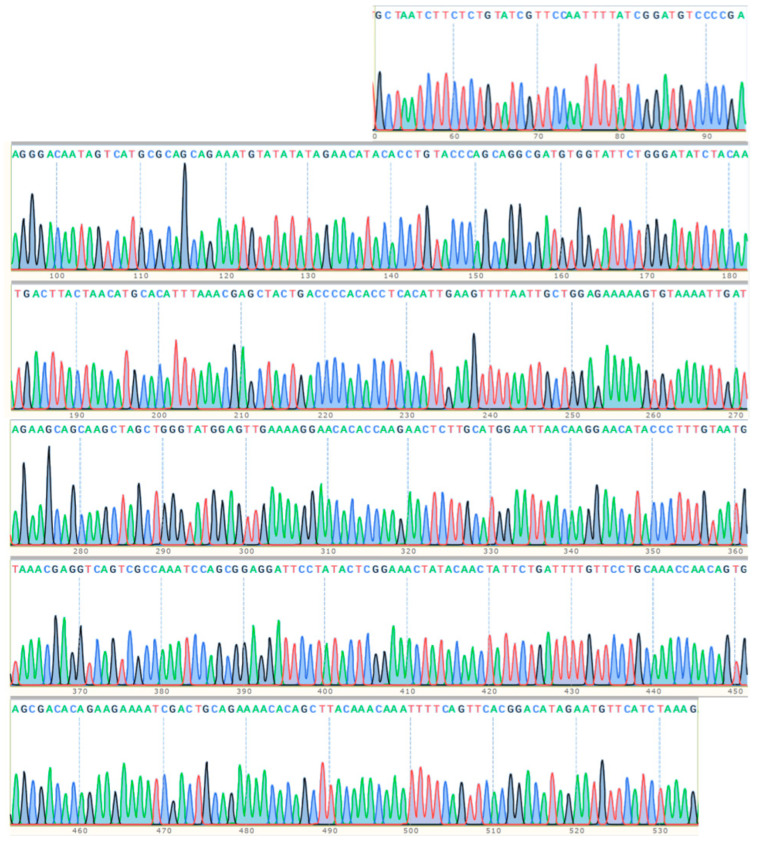
The Sanger sequencing results of the target region of the *LusPDS* gene in the leaves of the flax variety Longya 10 revealed a sequence length of 926 bp.

In green plants, *Phytoene desaturase* (*PDS*) is a key enzyme in carotenoid biosynthesis, and its knockout mutants exhibit an albino phenotype due to disrupted chlorophyll synthesis [36]. Leveraging this visible phenotypic marker, *PDS* has been widely adopted as a target gene to evaluate CRISPR/Cas9 editing efficiency across diverse plant species [25,37,38]. In flax, a pair of sgRNAs targeting the first exon of *Phytoene desaturase* (*LusPDS*, *Lus10021967*) were designed using the CRISPOR platform (https://crispor.gi.ucsc.edu/) (accessed on 3 April 2025) (Figure 5a). To ensure the absence of genotype-specific SNPs that might interfere with editing, the target region of *LusPDS* in the flax cultivar Longya 10 was amplified and validated by Sanger sequencing, confirming alignment with the reference sequence (Figure A1).

**Figure A2:**
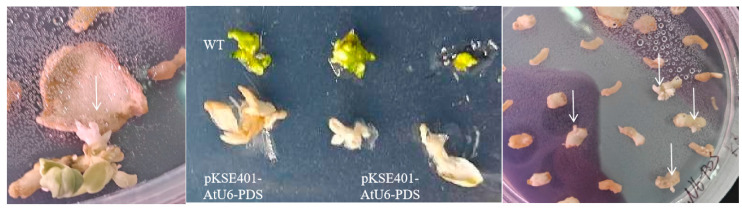
Transgenic plants transformed with pKSE401-AtU6-PDS and pKSE401-LuU6-PDS.

To assess the ability of endogenous flax promoters to drive sgRNA expression, the *Arabidopsis AtU6-26* promoter in the pKSE401 vector was replaced with the truncated flax *Lu14U6-4-5P* (342 bp) promoter using Gibson assembly (Figure 5b). Sequencing confirmed the successful construction of CRISPR/Cas9 vectors (pKSE401-PDS) expressing sgRNA1 and sgRNA2 under the control of either *Lu14U6-4-5P* or *AtU6-26* (Figure 5c). These vectors, designated pKSE401-LuU6-PDS and pKSE401-AtU6-PDS, were introduced into flax hypocotyl embryogenic cells via Agrobacterium-mediated transformation. Following selection on LU3 medium, 32.5% of hypocotyls transformed with pKSE401-LuU6-PDS and 34.9% with pKSE401-AtU6-PDS produced albino shoots. PCR using Cas9-specific primers confirmed the integration of exogenous T-DNA in kanamycin-resistant cells, validating successful CRISPR system delivery (Table 2, Figure 5d,e and Figure A2).

### 2.5. The LuU6 Promoter Demonstrates Higher Editing Efficiency in CRISPR/Cas9-Mediated Gene Editing in Flax Compared to the AtU6 Promoter

**Figure 6:**
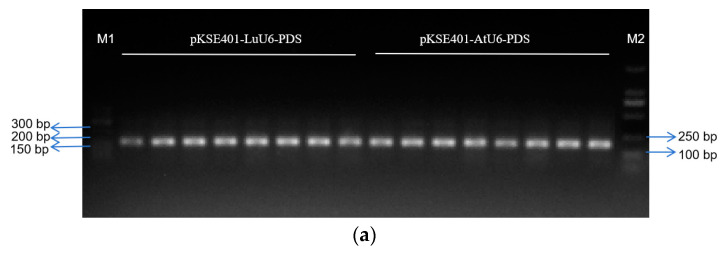

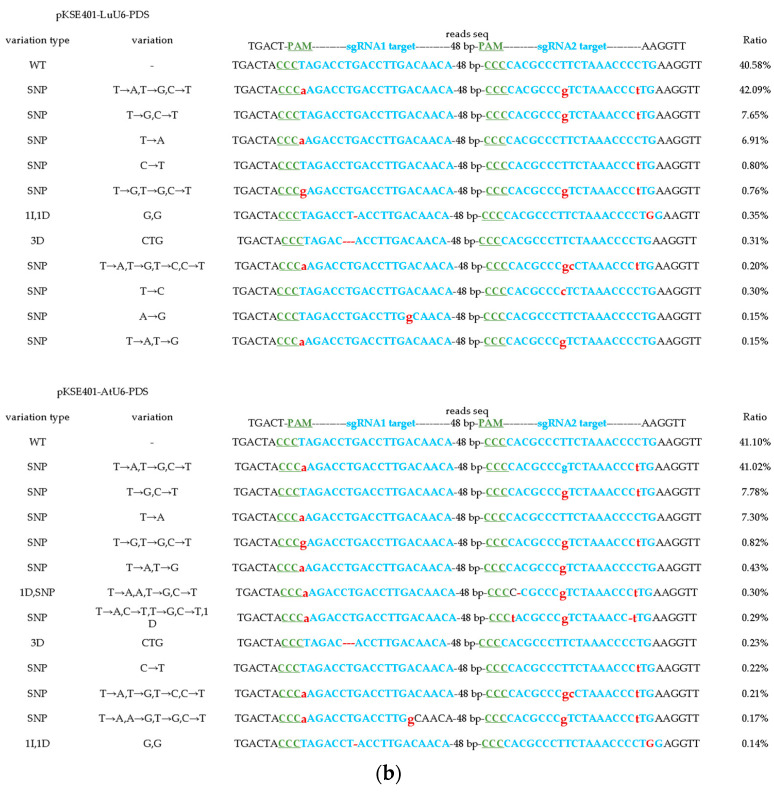
Comparative analysis of CRISPR/Cas9-mediated genome editing efficiency in flax using endogenous *Lu14U6-4-5P* and heterologous *AtU6-26* promoter-driven vectors. (**a**) Amplification of sgRNA1 and sgRNA2 editing fragments in DNA from albino shoots of flax hypocotyls transformed with pKSE401-LuU6-PDS and pKSE401-AtU6-PDS vectors; (**b**) summary of mutation types retained after filtering non-informative mutations outside sgRNA target regions. Variation Type: categories include single-base deletions (1D), single-base insertions (1I), and single nucleotide polymorphisms (SNPs), all located within 100 bp of the primer. Variation: specific nucleotide changes within the 100 bp region flanking the primer. Ratio: proportion of each mutation type relative to the total mutations in the sample (number of mutated sequences identified via high-throughput sequencing, corresponding to the number of T-vector single clones analyzed). Reads Sequence: sequencing reads with lowercase letters indicating mutation positions.

High-throughput sequencing platforms, such as Hi-TOM [39], have emerged as powerful tools for precisely quantifying CRISPR/Cas9 editing efficiencies, particularly in complex plant genomes where multiplexed sgRNA systems are employed [40]. These platforms enable the detection of diverse mutation types, including single-nucleotide polymorphisms (SNPs) and small insertions/deletions (indels), with high resolution [38]. To evaluate the editing efficacy of the *Lu14U6-4-5P*- and *AtU6-26*-driven CRISPR/Cas9 systems in flax, sequences containing sgRNA1 and sgRNA2 editing sites were amplified (Figure 6a) and analyzed using the Hi-TOM platform. Both vectors induced mutations in over 40% of transgenic cells across all samples. In pKSE401-LuU6-PDS-transformed cells, SNP mutations (e.g., T → A, T → G, and C → T substitutions) accounted for ≥36.60% of total mutations, while pKSE401-AtU6-PDS-transformed cells exhibited ≥34.91% SNPs (Table A2 and Table A3). Notably, indels were observed at specific loci: pKSE401-LuU6-PDS samples showed 2.73% deletions/insertions at G sites (Sample 5) and 2.34% deletions at CTG sites (Sample 6), whereas pKSE401-AtU6-PDS samples displayed 2.41% SNPs with G deletions (Sample 5), 2.18% SNPs with C deletions (Sample 6), and minor indels (1.03–1.80%) in other samples (Table A3).

**Figure A3:**
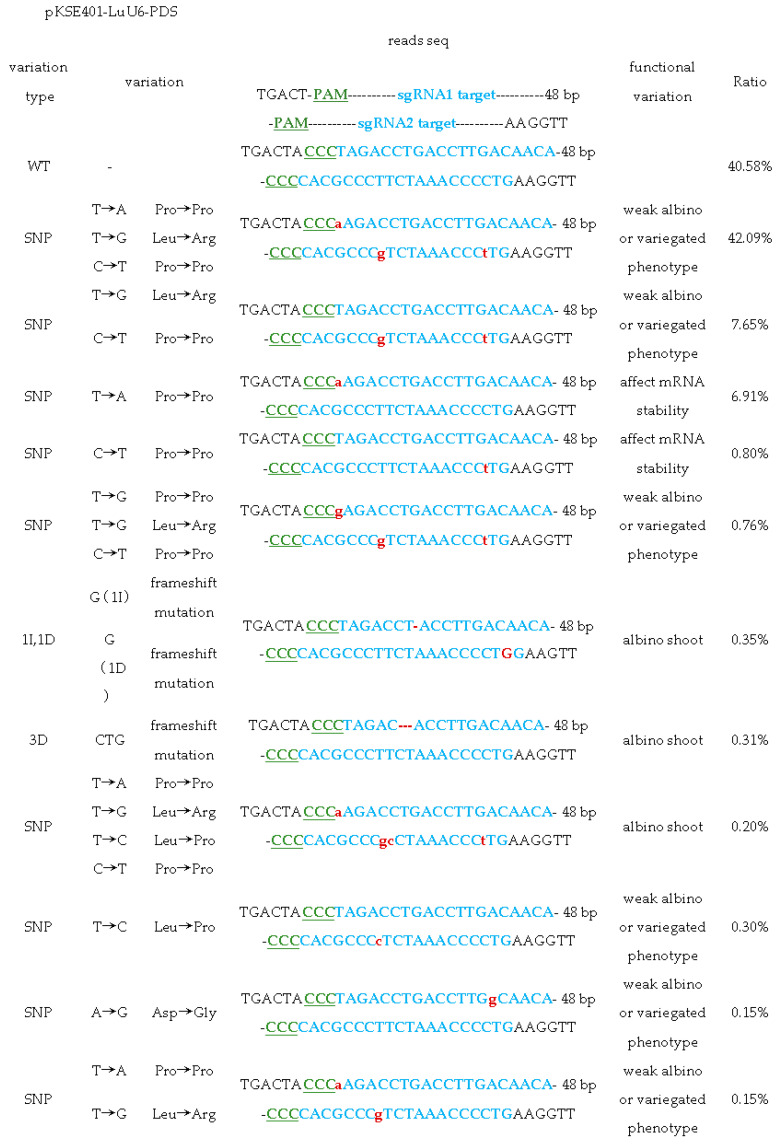
pKSE401-LuU6-PDS-mediated amino acid mutation types in *LusPDS* gene of transgenic flax regenerated shoots.

**Figure A4:**
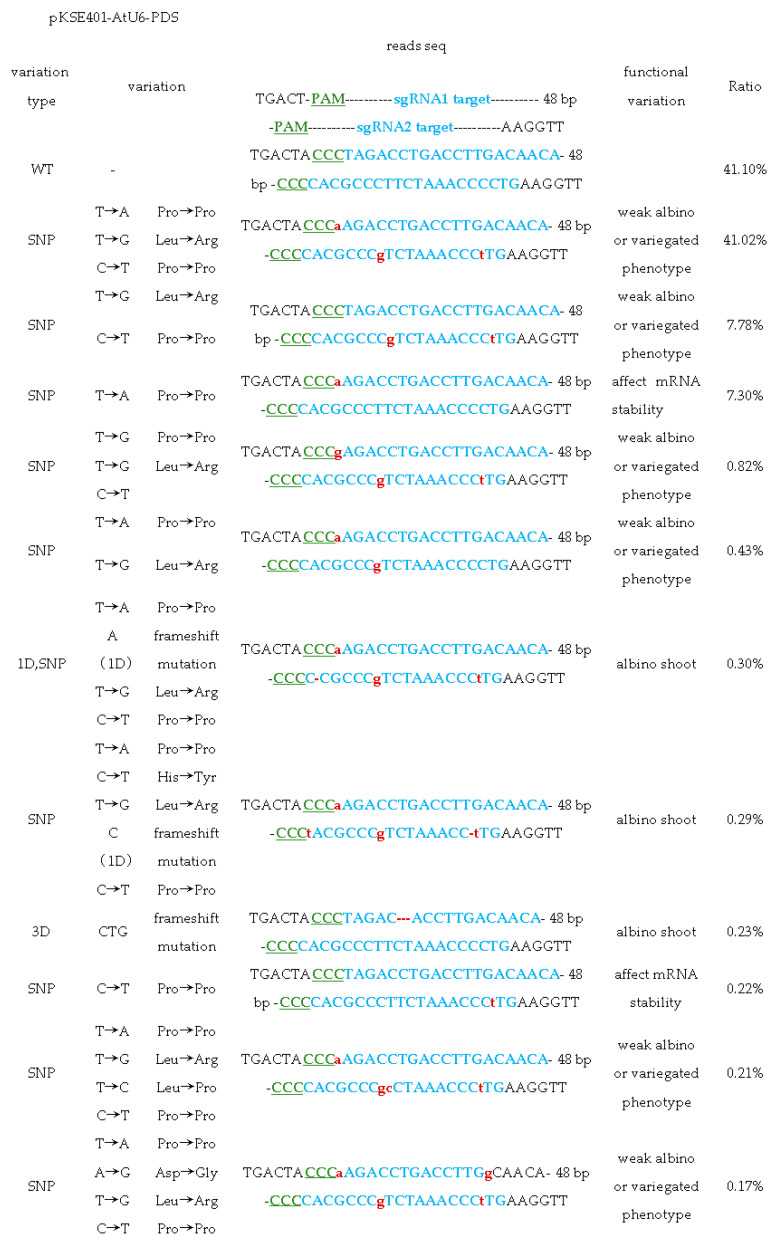
pKSE401-AtU6-PDS-mediated amino acid mutation types in *LusPDS* gene of transgenic flax regenerated shoots.

The genetic variations generated by CRISPR/Cas9 editing are primarily driven by the repair properties of non-homologous end joining (NHEJ). We hypothesize that base deletions may be directly associated with the preferential repair tendency of NHEJ in T/A-rich regions [41]. For example, deletion of “A” in codon “CAC”, insertion of “G” between “T” and “G” in codon “CTG”, deletion of “CTG”, or deletion of “G” result in frameshift mutations that disrupt the reading frame of *LusPDS*, completely destroying the catalytic domain of the PDS protein and ultimately leading to carotenoid biosynthesis disruption and albino phenotypes (Figure 5d, Figure A3 and Figure A4). Notably, microhomology near the target site, such as repetitive sequences, may guide precise 3 bp deletions [42]. Single-base mutations account for ≥34.91% of editing events, among which missense mutations reduce enzymatic activity, resulting in weakly albino regenerated shoots. For instance, the A → G mutation in codon “GAC” causes aspartic acid to change to glycine, while T → G or T → C substitutions in codon “CTT”, or TT → GC replacements, lead to leucine (nonpolar) → arginine (positively charged) or proline (rigid structure). Additionally, the first “C” in “CAC” mutating to “T” converts histidine to tyrosine. These missense mutations (Figure A3 and Figure A4) disrupt the hydrophobic core or substrate-binding site of the PDS enzyme, reducing catalytic efficiency and decreasing carotenoid synthesis. Consequently, regenerated shoots exhibit weakly albino phenotypes and slow growth.

We analyzed all missense and frameshift mutations (including insertions and deletions) in samples tested for editing efficiency (Table A2). Over 81% of albino or weakly albino regenerated shoots were associated with missense mutations, while 1.8–4.6% of frameshift mutations caused by insertions or deletions were observed in two transgenic samples, each showing 2–3 albino regenerated shoots. These nucleotide mutations, deletions, and insertions ultimately alter amino acids, disrupting hydrogen bond formation, enzymatic catalysis, or structural stability, consistent with the albino or weakly albino phenotypes observed in transgenic regenerated shoots. Aggregate analysis revealed that the *Lu14U6-4-5P*-driven system achieved a 59.42% editing frequency at the *LusPDS* locus, slightly higher than the *AtU6-26*-driven system (58.90%) (Figure 6b). The marginal difference may stem from variability inherent to flax genetic transformation methods or experimental materials. Nevertheless, the endogenous Lu14U6-4-5P promoter enabled efficient genome editing in flax, demonstrating its utility for CRISPR/Cas9 applications in this species. While indel frequencies were comparable between the two systems, the majority of mutations in flax were SNPs, consistent with findings in citrus protoplasts transiently transfected with Cas9-PDS vectors [40]. This parallels reports in lettuce, where endogenous *LSU6-10* outperformed *AtU6-26* in driving CRISPR/Cas9 activity [38]. The modest yet consistent superiority of *Lu14U6-4-5P* underscores the importance of species-specific promoters in enhancing editing precision and efficiency. These results validate the efficacy of the flax endogenous *Lu14U6-4-5P* promoter in CRISPR/Cas9-mediated genome editing, providing a robust framework for optimizing gene editing tools in crops with complex genetic backgrounds.

## 3. Discussion

As an RNA-guided DNA endonuclease system, the activity of sgRNA and the ex-pression levels of the sgRNA/Cas9 complex are critical determinants of CRISPR/Cas9-mediated genome editing efficiency [43,44]. Employing species-specific promoters is an effective strategy to enhance plant genome engineering efficiency [25,45]. To date, eukaryotic U3 and U6 promoters isolated from rice and *Arabidopsis* have been widely used in monocots and dicots, respectively [11,46]. Both reporter systems (e.g., luciferase and GUS) are widely used for transcriptional activity validation. For instance, GUS reporter assays demonstrated that the transcriptional level of the U6-4 SnRNA promoter-controlled GUS fragment in *Arabidopsis* was higher than those controlled by U6-5 and U6-6 promoters, revealing the functional activity of three newly identified U6SnRNA genes [47]. In flax, the AtU6 and 35S promoters are typically utilized to drive sgRNA and Cas9 expression in genome editing [48,49,50]. In this study, we identified four candidate flax U6 promoters through sequence alignment and cis-acting element analysis. Due to the high conservation of U6 snRNA sequences, the transcriptional activity of these promoters in our study was evaluated using a dual-luciferase (LUC) reporter system [51], which provides superior quantitative accuracy, sensitivity, and experimental efficiency, enabling rapid and precise comparison of promoter activities—critical for functional validation of conserved regulatory elements [52]. Among the candidates, *Lu14U6-4* exhibited the highest transcriptional activity.

However, large promoter sequences can increase vector size, posing challenges for multiplex sgRNA expression [53,54]. To address this, we truncated *Lu14U6-4* from the 5′ end and constructed truncated variants. Transient transformation assays revealed that the 342 bp *Lu14U6-4-5P* fragment retained the highest transcriptional activity. Notably, shorter promoters (138 bp and 142 bp) retained partial activity, suggesting that core regulatory elements are preserved, while reduced activity may reflect the absence of auxiliary enhancers [55], a phenomenon also observed in apple [34]. Moreover, in castor, a 300 bp U6 promoter is sufficient to activate gene expression [26].

To evaluate *Lu14U6-4-5P* in genome editing, we replaced the *AtU6-26* promoter in the pKSE401 vector with *Lu14U6-4-5P* and targeted *Phytoene desaturase* (*LusPDS*) in flax. Transgenic lines exhibited high mutation frequencies, with *Lu14U6-4-5P*-driven constructs achieving 59.42% efficiency, marginally surpassing *AtU6-26*-driven constructs (58.90%). Similar studies in citrus and lettuce demonstrated superior performance of endogenous U6 promoters over heterologous counterparts [38,40]. The pre-dominant mutations were single-nucleotide polymorphisms (SNPs) and small insertions/deletions (indels), consistent with typical CRISPR/Cas9 outcomes [56]. The transcriptional activity of *PqU6-7PA* promoter (283 bp) was 1.59 times that of *AtU6-P* promoter (292 bp) when the recipient material was *P. quinquefolium* callus [57]. These results underscore that species-specific promoters enhance editing efficiency in native systems.

sgRNAs recruit Cas9 to induce site-specific double-strand breaks (DSBs) [58], typically repaired via error-prone non-homologous end joining (NHEJ), leading to frameshift mutations and loss-of-function alleles. Rational sgRNA design targeting conserved regions, such as the first exon of *LusPDS* in this study, minimizes off-target effects while maximizing editing efficiency. For instance, in wheat, sgRNAs targeting conserved exons achieved uniform mutations across homoeologs [59], whereas in watermelon *ClCIPK17*, sgRNA1 induced widespread deletions while sgRNA5 showed no activity [60]. In flax, *LusPDS* editing primarily resulted in SNPs and minor indels, contrasting with alfalfa and asparagus, where indels reached 40–80% [61,62]. For instance, *B. distachyon* promoter sequences have been found to be functional in wheat, exhibiting high efficiency in gene transformation. This stands in contrast to promoters from other parts of rice and wheat, which have not demonstrated the same level of effectiveness in driving gene expression. [63]. This highlights the influence of species, target genes, sgRNA design, and transformation methods on editing outcomes.

The predominance of single-nucleotide mutations (≥34.91% SNPs) and frameshift deletions (e.g., 1 bp del) in our Hi-TOM data (Table A3) underscores the efficiency of the *LuU6* and *AtU6* promoters in driving sgRNA expression [11]. Critically, the albino phenotype observed in edited flax tissues (Figure 5d) directly correlates with frameshift mutations (1 bp del) in *LusPDS*, which disrupt the enzyme’s ability to catalyze phytoene desaturation [36]—a bottleneck step in carotenoid biosynthesis. Conversely, the rare 3 bp deletions (e.g., in CTG motifs) likely remove non-essential residues, explaining the occasional survival of weakly pigmented callus. These findings mirror reports in *Arabidopsis*, where in-frame deletions in *PDS* caused variegated phenotypes rather than complete albinism [64]. In summary, we identified and characterized multiple flax U6 promoters, truncating *Lu14U6-4* to optimize its length. The 342 bp *Lu14U6-4-5P* variant demonstrated superior transcriptional activity and editing efficiency, providing a foundation for developing tailored flax genome editing tools. The use of endogenous promoters like *Lu14U6-4-5P* offers a promising strategy to enhance CRISPR/Cas9 efficiency in flax and other crops, advancing functional genomics and precision breeding efforts.

## 4. Materials and Methods

### 4.1. Plant Materials and Treatment

The plant materials used for extracting flax genomic DNA and RNA were selected from the seeds of “Longya 10”, one of the primary cultivated oil flax varieties. The seeds were grown under controlled conditions with a photoperiod of 16 h of light and 8 h of darkness, at a temperature of 23 (±2) °C. After 3–4 weeks of growth, healthy flax leaves were harvested. Genomic DNA was extracted from the leaves using the CTAB method [65], while total RNA was isolated using the TransZol Up Plus RNA Kit.

To obtain transiently transformed flax embryogenic callus, plump seeds were surface-sterilized and then longitudinally sectioned with a scalpel. The seeds were inoculated onto MS medium supplemented with 1.6 mg/L 2,4-D and cultured in the dark for 20 days. The embryogenic callus, characterized by a compact texture and nodular protrusions, was transferred to a subculture medium with the same composition as the induction medium for further proliferation.

For transient transformation of *Nicotiana benthamiana*, seeds were evenly sown in a soil mixture composed of potting soil, vermiculite, and perlite (3:1:1, *v*/*v*/*v*). The plants were grown in a greenhouse at 25 °C under a 16 h light/8 h dark cycle.

### 4.2. Cloning, Truncation, and Construction of Dual-Luciferase Reporter Gene Expression Vectors for Flax LuU6 Promoter

To identify candidate U6 snRNAs in flax, a 120 bp conserved U6 snRNA sequence from Arabidopsis thaliana was used to search the flax reference genome. The candidate flax U6 promoter sequences were aligned with the *AtU6-26* promoter from Arabidopsis using DNAMAN, and sequences with low similarity were excluded. The promoter regions of four different flax U6 snRNAs and their upstream sequences were predicted using the online tool PlantCARE (https://bioinformatics.psb.ugent.be/webtools/plantcare/html/) (accessed on 3 April 2025) to confirm the candidate U6 promoter sequences. The identified candidate sequences were further analyzed for promoter elements using PlantCARE [66], and the results were visualized using TBtools.

The candidate flax U6 promoter sequences were used as templates for PCR amplification of the four U6 promoters. The amplified products were ligated and transformed. Positive clones were screened using colony PCR, and correctly identified clones were inoculated into liquid LB medium containing 50 mg/L kanamycin and cultured overnight at 37 °C with shaking at 180 rpm. Plasmid extraction was performed, and the plasmids were sequenced and analyzed for sequence alignment.

The verified plasmids containing the flax U6 promoters and the pGreenII0800-LUC vector (Wuhan Transduction Biology, Figure 1a) were double-digested with the same restriction enzymes, and the target bands were purified. The ligation products were heat-shock transformed into *E. coli* DH5α. Positive clones were selected, inoculated into liquid LB medium, and cultured overnight at 37 °C with shaking at 180 rpm. Plasmids were extracted and verified by double digestion. The correctly identified plasmids were then transformed into Agrobacterium tumefaciens GV3101 competent cells using the liquid nitrogen method.

To investigate the influence of sequence length on the transcriptional activity of *Lu14U6-4*, the *Lu14U6-4* promoter sequence was analyzed using the PLACE and PlantCARE online tools for promoter element prediction. Based on the results of the sequence element analysis, the *Lu14U6-4* promoter was truncated at the 5′ end according to the distribution of CAAT-box motifs. Primers were designed using Primer Premier 5.0, and high-fidelity PCR amplification was performed using Prime STAR MAX DNA Polymerase to generate truncated versions of *Lu14U6-4*, including *Lu14U6-4-1P*, *Lu14U6-4-2P*, *Lu14U6-4-3P*, *Lu14U6-4-4P*, and *Lu14U6-4-5P*. These truncated promoter fragments were subsequently cloned into dual-luciferase reporter gene plant expression vectors for further functional analysis.

### 4.3. Transient Transformation of Flax Callus and Nicotiana benthamiana

An optimized Agrobacterium-mediated transient transformation method was employed for flax [67,68]. A 10-fold diluted bacterial colony mixture was used as the template for colony PCR. Positive Agrobacterium colonies were selected and inoculated into liquid LB medium supplemented with 50 mg/L kanamycin and 20 mg/L Rifampicin for expansion. Bacterial cells were collected and resuspended in a buffer solution (10 mM MgCl_2_, 150 μM acetosyringone, 10 mM MES) to an OD600 of 0.8. The OD600 values of all bacterial suspensions were adjusted to ensure consistency. The mixed Agrobacterium suspension was incubated at 28 °C for 3 h. Flax callus tissues were immersed in the infection solution and co-cultured at 28 °C with shaking at 180 rpm for 30 min. The infection solution was discarded, and the callus tissues were rinsed with sterile water until clean, then dried with filter paper. The callus tissues were placed on callus induction medium and co-cultured in the dark at 28 °C for 4 days.

For *Nicotiana benthamiana* transformation, the Agrobacterium suspension was centrifuged, and the bacterial pellet was resuspended in the infection solution. After standing at room temperature, 300 μL of the mixed bacterial suspension was drawn into a 1 mL syringe. A small wound was carefully made on the underside of the tobacco leaf using a needle, and the bacterial suspension was slowly injected into the leaf, allowing it to absorb completely. Three biological replicates were performed. The treated plants were kept in the dark for 12 h, followed by 48 h of light exposure.

### 4.4. Dual-Luciferase Reporter Gene Assay for Flax LuU6 Promoter

A 150 μg/mL sodium luciferin solution was evenly applied to the transiently transformed flax callus tissues and tobacco leaves. After allowing the reaction to proceed for 2 min, images were captured using a fluorescence-labeled organism bioimaging instrument. Protein extracts from both non-transformed and transformed flax callus tissues and tobacco leaves were prepared using the Beyotime Dual-Lumi™ II Dual-Luciferase Reporter Gene Assay Kit (RG089S). The enzymatic activities of LUC and REN were measured, and the results were expressed as the ratio of LUC to REN (LUC/REN).

All the experiments were repeated four times. The experimental data processing and mapping were completed using Excel 2020, Graphpad Prism 9.5, IBM SPSS statistics 20.0, and TBtools. All values were expressed as mean ± standard error. The mean was compared using analysis of variance (ANOVA) followed by Duncan’s honestly significant difference test. Significant differences among groups are indicated by different lowercase letters (*p* < 0.05).

### 4.5. Construction of CRISPR/Cas9-Mediated Gene Editing Vectors in Flax

The transcript sequence of *Phytoene desaturase* (*LusPDS*, *Lus10021967*) in flax was obtained from the Phytozome database (https://phytozome-next.jgi.doe.gov/) (accessed on 3 April 2025). A pair of sgRNAs targeting *LusPDS*, sgRNA1 and sgRNA2, both located in the first exon, were designed using the CRISPOR web server (https://crispor.gi.ucsc.edu/) (accessed on 3 April 2025) [69]. To ensure the absence of potential genotype SNPs that might affect genome editing due to mismatches, genomic DNA was extracted from leaves of the flax cultivar Longya 10. The target region was amplified by PCR and validated by Sanger sequencing. A CRISPR/Cas9 vector, pKSE401-PDS, containing sgRNA1 and sgRNA2, was constructed using a modified method [70].

To facilitate the replacement of the *Arabidopsis AtU6* promoter in the pKSE401 vector with the endogenous flax U6 promoter, a LacZ cassette flanked by BsaI restriction sites was amplified from the plasmid pICH47742 of the MoClo Plant Parts Kit (Addgene distributor, Beijing, China) [71] and inserted into the pKSE401 vector using Gibson assembly [72]. By performing a BLAST search using the *Arabidopsis* U6 promoter (AtU6-29), which contains characteristic upstream sequence element (USE) motifs and a TATA box, five candidate flax promoters were identified. The promoter with the highest activity, as determined by the dual-luciferase assay, was selected. This promoter, along with a polycistronic tRNA-gRNA (PTG) fragment containing gRNA1/2 (tRNA-gRNA1-tRNA-gRNA2) [8], was inserted into the modified pKSE401 vector to construct the pKSE401-LuU6-PDS and pKSE401-AtU6-PDS vectors. The vectors were transformed into *E. coli* DH5α via heat shock, and the correctly sequenced plasmids were introduced into *Agrobacterium tumefaciens* GV3101 competent cells using the liquid nitrogen method.

### 4.6. Plant Vector Construction

Seeds of flax (variety: Longya) were surface sterilized by treatment with 75% ethanol for 3 min, followed by three washes with sterile water. Subsequently, the seeds were treated with 0.1% sodium hypochlorite for 10 min and washed nine times with sterile water. The sterilized seeds were then sown on LU1 germination medium and incubated at 25 °C in the dark for 3–5 days. After germination, the seedlings were transferred to conditions with 8 h of light for further growth.

Sterile flax seedlings aged 5–10 days were used for transformation. Hypocotyls were cut into 3–5 mm segments and immersed in an *Agrobacterium tumefaciens* suspension with an OD600 of 0.6 for 30 min. After drying, the explants were placed on LU2 co-culture medium and incubated at 25 °C for 2–3 days. The explants were then washed with a 500 mg/L carbenicillin solution for 30 min and transferred to LU3 selection medium, with subculturing every two weeks [73].

Once albino shoots emerged from the hypocotyls, genomic DNA was extracted from the shoots using the CTAB method [66]. The target gene fragments were amplified using the Hi-TOM-*LusPDS* F and Hi-TOM-*LusPDS* R primer pairs (Table A1). The PCR products were cloned into a T-vector and directly sequenced using the Hi-TOM platform (China National Rice Research Institute, Chinese Academy of Agricultural Sciences, Hangzhou, Zhejiang, China) [39].

## 5. Conclusions

In this study, we systematically identified and characterized endogenous U6 promoters in flax to optimize CRISPR/Cas9-mediated genome editing efficiency. Through sequence alignment and cis-element analysis, four candidate U6 promoters were identified, with *Lu14U6-4* exhibiting the highest transcriptional activity in dual-luciferase assays. Truncation of *Lu14U6-4* from the 5′ end revealed that a 342 bp fragment (*Lu14U6-4-5P*) retained optimal transcriptional activity while minimizing vector size, addressing challenges associated with multiplex sgRNA delivery. Replacement of the heterologous *AtU6-26* promoter in the pKSE401 vector with *Lu14U6-4-5P* resulted in a marginally higher editing efficiency (59.42% vs. 58.90%) when targeting *Phytoene desaturase* (*LusPDS*), predominantly generating single-nucleotide polymorphisms (SNPs) and minor indels. These findings align with studies in citrus and lettuce, where endogenous U6 promoters outperformed heterologous counterparts, underscoring the importance of species-specific regulatory elements in enhancing editing precision.

The success of this strategy highlights the critical role of promoter optimization in CRISPR/Cas9 workflows, particularly for species like flax with transformation efficiency constraints. By integrating endogenous promoters such as *Lu14U6-4-5P*, we provide a robust framework for developing tailored genome editing tools in flax, advancing functional genomics and precision breeding. Future work should explore multiplexed sgRNA delivery and broader applications of these promoters in other agronomically important traits, further bridging the gap between CRISPR technology and crop improvement.

## Figures and Tables

**Table 1:** The chromosomal localization and genome BLAST results of flax U6 snRNA.

Gene Name	Chromosome Location	E-Value
*Lu14U6-1*	Chr14: 10801770…10801880	3.00 × 10^−49^
*Lu14U6-2*	Chr14: 10810787…10810897	3.00 × 10^−49^
*Lu14U6-3*	Chr14: 10613237…10613349	1.00 × 10^−48^
*Lu14U6-4*	Chr14: 13709531…13709640	1.00 × 10^−48^
*Lu14U6-5*	Chr14: 13672928…13673036	4.00 × 10^−48^
*Lu14U6-6*	Chr14: 13685856…13685964	4.00 × 10^−48^
*Lu14U6-7*	Chr14: 5535449…5535484	6.00 × 10^−6^
*Lu13U6-1*	Chr13: 17095955…17096063	4.00 × 10^−48^
*Lu13U6-2*	Chr13: 14893350…14893457	1.00 × 10^−47^
*Lu13U6-3*	Chr13: 18154867…18154981	2.00 × 10^−46^
*Lu13U6-4*	Chr13: 14891387…14891491	6.00 × 10^−46^

**Table 2:** Editing frequency of CRISPR/Cas9-mediated *LusPDS* gene editing in transgenic flax hypocotyls.

Transformation Vector	Number of Transgenic Flax Hypocotyls	Total Number of Germinated Shoots	Number of Albino Shoots	Frequency of Albino Shoot Emergence
pKSE401-LuU6-PDS	80	45	26	32.5%
pKSE401-AtU6-PDS	83	46	29	34.9%

## Data Availability

The plant materials are available from the corresponding author (Li-Qiong Xie) upon reasonable request.

## Appendix A

**Table A1:** Primer sequence information.

Primer Name	Sequence (5′-3′)	Experiment
M13-F	GTAAAACGACGGCCAGT	Universal Primers for Vector
M13-R	CAGGAAACAGCTATGAC	
pGreenII-LUC-F	GTTTTCCCAGTCACGACGTT	Universal Primers for pGreenII0800-LUC Vector
pGreenII-LUC-R	GCCTTATGCAGTTGCTCTCC	
*Lu13U6-1*F	GGGGTACCCCCTAAAGATTTGGGGGTCACT	Cloning of Candidate U6 Promoter Fragments
*Lu13U6-1*R	CGGGATCCAGGGGCCATGCTAATCTTCTCT	
*Lu13U6-3*F	GGGGTACCCGAGAACAAACCTCTAGCCG	
*Lu13U6-3*R	CCCAAGCTTCTTGCGCAGGGGCCAT	
*Lu14U6-3*F	GGGGTACCTCCCCCTGGGTTAGTAGCAATA	
*Lu14U6-3*R	CCCAAGCTTAGGGGCCATGCTAATCTTCT	
*Lu14U6-4*F	GGGGTACCTGGGGTGCACAAACTATAGCG	
*Lu14U6-4*R	CCCAAGCTTAGGGGCCATGCTAATCTTCTC	
SK-R	TCTAGAACTAGTGGATC	Colony PCR Screening
*Lu14U6-4-1p*F *Lu14U6-4-2p*F *Lu14U6-4-3p*F	TCGACGGTATCGATAAGCTTAGGGGCCATGCTAATCTTCTCT	Cloning and Detection of Truncated U6 Promoter Fragments
*Lu14U6-4-1p*R	GCGGCCGCTCTAGAACTAGTCAAATTCAAAAATATATACA	
*Lu14U6-4-2p*R	GCGGCCGCTCTAGAACTAGTATTGTAGATATCCCAGAATACC	
*Lu14U6-4-3p*R	GCGGCCGCTCTAGAACTAGTTAGATATCCCAGAATACCAC	
*Lu14U6-4-4p*F *Lu14U6-4-5p*F	TCGAGGTCGACGGTATCGATAAGCTTAGGGGCCATGCTAATCTTCT	
*Lu14U6-4-4p*R	GCGGCCGCTCTAGAACTAGTGGATCCATTGTTTGATTGAATGGAAC	
*Lu14U6-4-5p*R	GCGGCCGCTCTAGAACTAGTGGATCCATTTGGCGACTGACCTCGTT	
M13F (47)	CGCCAGGGTTTTCCCAGTCACGAC	Detection of Truncated U6 Promoter Fragments
*LusPDS* F	TTCAGGGGAAGTGACTCTCTCA	Detection of the 926 bp *LusPDS* Fragment in the Genomic DNA of Longya 10 Leaves
*LusPDS* R	TCGTACCAGTCTCCATCCTC	
TDNA-*LusPDS* F1	CCCGCCAATATATCCTGTCAAAC	Detection of Exogenous T-DNA Insertion in Cas9 Editing Vectors
TDNA-*LusPDS* R1	CCCTAGACCTGACCTTGACAACA	
Hi-TOM-*LusPDS* F	ggagtgagtacggtgtgcGTGTTGTGCGTTGACTACCC	Hi-TOM Assay
Hi-TOM-*LusPDS* R	gagttggatgctggatggCCTGCACCAGCAATAACAAC	

**Table A2:** CRISPR/Cas9-mediated editing of *LusPDS* leads to albino phenotypes.

Sample Names	Proportions of Major Mutation Types			Mutant Phenotype
	Missense Mutation	Synonymous Mutation	Frameshift Mutation	
pKSE401-LuU6-PDS-ref 1	85.57%	14.43%	0	albino shoot
pKSE401-LuU6-PDS-ref 2	88.19%	11.81%	0	albino shoot
pKSE401-LuU6-PDS-ref 3	89.55%	10.45%	0	slightly albino shoots
pKSE401-LuU6-PDS-ref 4	87.04%	12.96%	0	slightly albino shoots
pKSE401-LuU6-PDS-ref 5	86.98%	8.52%	4.50%	albino shoot
pKSE401-LuU6-PDS-ref 6	83.23%	12.51%	4.26%	albino shoot
pKSE401-LuU6-PDS-ref 7	83.57%	16.43%	0	slightly albino shoots
pKSE401-LuU6-PDS-ref 8	86.44%	13.56%	0	slightly albino shoots
pKSE401-AtU6-PDS-ref 1	89.28%	10.72%	0	slightly albino shoots
pKSE401-AtU6-PDS-ref 2	87.70%	12.31%	0	slightly albino shoots
pKSE401-AtU6-PDS-ref 3	88.25%	11.75%	0	slightly albino shoots
pKSE401-AtU6-PDS-ref 4	85.95%	14.05%	0	slightly albino shoots
pKSE401-AtU6-PDS-ref 5	83.56%	11.82%	4.62%	albino shoot
pKSE401-AtU6-PDS-ref 6	83.98%	16.02%	0	slightly albino shoots
pKSE401-AtU6-PDS-ref 7	81.92%	16.28%	1.80%	albino shoot
pKSE401-AtU6-PDS-ref 8	85.2%	11.43%	3.31%	albino shoot

**Table A3:** After excluding non-informative mutations outside the sgRNA target sites for each sample, a summary of the various types of mutations that remain is presented below.

pKSE401-LuU6-PDS					
Sort	Reads Number	Ratio	Variation Type	Variation	Reads Seq
001-ref					TGACT-**PAM**----------**sgRNA1 target**----------48 bp-**PAM**----------**sgRNA2 target**----------AAGGTT
1	1494	38.48%	WT	-	TGACTA**CCCTAGACCTGACCTTGACAACA**-48 bp-**CCCCACGCCCTTCTAAACCCCTG**AAGGTT
2	1555	40.05%	SNP	T → A, T → G, C → T	TGACTA**CCCa**AGACCTGACCTTGACAACA-48 bp-**CCC**CACGCCC**g**TCTAAACCC**t**TGAAGGTT
3	305	7.85%	SNP	T → A	TGACTA**CCCa**AGACCTGACCTTGACAACA-48 bp-**CCC**CACGCCCTTCTAAACCCCTGAAGGTT
4	251	6.46%	SNP	T → G, C → T	TGACTA**CCC**TAGACCTGACCTTGACAACA-48 bp-**CCC**CACGCCC**g**TCTAAACCC**t**TGAAGGTT
5	46	1.18%	SNP	T → G, T → G, C → T	TGACTA**CCCg**AGACCTGACCTTGACAACA-48 bp-**CCC**CACGCCC**g**TCTAAACCC**t**TGAAGGTT
6	44	1.13%	SNP	T → A, T → G	TGACTA**CCCa**AGACCTGACCTTGACAACA-48 bp-**CCC**CACGCCC**g**TCTAAACCCCTGAAGGTT
7	40	1.03%	SNP	C → T	TGACTA**CCC**TAGACCTGACCTTGACAACA-48 bp-**CCC**CACGCCCTTCTAAACCC**t**TGAAGGTT
002-ref					
1	1645	42.58%	WT	-	TGACTA**CCCTAGACCTGACCTTGACAACA**-48 bp-**CCCCACGCCCTTCTAAACCCCTG**AAGGTT
2	1473	38.13%	SNP	T → A, T → G, C → T	TGACTA**CCCa**AGACCTGACCTTGACAACA-48 bp-**CCC**CACGCCC**g**TCTAAACCC**t**TGAAGGTT
4	262	6.78%	SNP	T → A	TGACTA**CCCa**AGACCTGACCTTGACAACA-48 bp-**CCC**CACGCCCTTCTAAACCCCTGAAGGTT
3	222	5.75%	SNP	T → G, C → T	TGACTA**CCC**TAGACCTGACCTTGACAACA-48 bp-**CCC**CACGCCC**g**TCTAAACCC**t**TGAAGGTT
5	54	1.40%	SNP	T → G, T → G, C → T	TGACTA**CCCg**AGACCTGACCTTGACAACA-48 bp-**CCC**CACGCCC**g**TCTAAACCC**t**TGAAGGTT
003-ref					
1	1551	41.22%	WT	-	TGACTA**CCCTAGACCTGACCTTGACAACA**-48 bp-**CCCCACGCCCTTCTAAACCCCTG**AAGGTT
2	1501	39.89%	SNP	T → A, T → G, C → T	TGACTA**CCCa**AGACCTGACCTTGACAACA-48 bp-**CCC**CACGCCC**g**TCTAAACCC**t**TGAAGGTT
3	256	6.80%	SNP	T → G, C → T	TGACTA**CCC**TAGACCTGACCTTGACAACA-48 bp-**CCC**CACGCCC**g**TCTAAACCC**t**TGAAGGTT
4	205	5.45%	SNP	T → A	TGACTA**CCCa**AGACCTGACCTTGACAACA-48 bp-**CCC**CACGCCCTTCTAAACCCCTGAAGGTT
004-ref					
1	1451	37.52%	WT	-	TGACTA**CCCTAGACCTGACCTTGACAACA**-48 bp-**CCCCACGCCCTTCTAAACCCCTG**AAGGTT
2	1558	40.29%	SNP	T → A, T → G, C → T	TGACTA**CCCa**AGACCTGACCTTGACAACA-48 bp-**CCC**CACGCCC**g**TCTAAACCC**t**TGAAGGTT
3	277	7.17%	SNP	T → A	TGACTA**CCCa**AGACCTGACCTTGACAACA-48 bp-**CCC**CACGCCCTTCTAAACCCCTGAAGGTT
4	201	5.20%	SNP	T → G, C → T	TGACTA**CCC**TAGACCTGACCTTGACAACA-48 bp-**CCC**CACGCCC**g**TCTAAACCC**t**TGAAGGTT
5	60	1.55%	SNP	T → A, T → G, T → C, C → T	TGACTA**CCCa**AGACCTGACCTTGACAACA-48 bp-**CCC**CACGCCC**gc**CTAAACCC**t**TGAAGGTT
6	43	1.11%	SNP	T → G, T → G, C → T	TGACTA**CCCg**AGACCTGACCTTGACAACA-48 bp-**CCC**CACGCCC**g**TCTAAACCC**t**TGAAGGTT
005-ref					
1	1483	39.32%	WT	-	TGACTA**CCCTAGACCTGACCTTGACAACA**-48 bp-**CCCCACGCCCTTCTAAACCCCTG**AAGGTT
2	1542	40.88%	SNP	T → A, T → G, C → T	TGACTA**CCCa**AGACCTGACCTTGACAACA-48 bp-**CCC**CACGCCC**g**TCTAAACCC**t**TGAAGGTT
3	361	9.57%	SNP	T → G, C → T	TGACTA**CCC**TAGACCTGACCTTGACAACA-48 bp-**CCC**CACGCCC**g**TCTAAACCC**t**TGAAGGTT
4	195	5.17%	SNP	T → A	TGACTA**CCCa**AGACCTGACCTTGACAACA-48 bp-**CCC**CACGCCCTTCTAAACCCCTGAAGGTT
5	103	2.73%	1I,1D	G, G	TGACTA**CCC**TAGACCT**-**ACCTTGACAACA-48 bp-**CCC**CACGCCCTTCTAAACCCCT**G**GAaGTT
6	88	2.33%	SNP	T → C	TGACTA**CCC**TAGACCTGACCTTGACAACA-48 bp-**CCC**CACGCCC**c**TCTAAACCCCTGAAGGTT
006-ref					
1	1601	41.64%	WT	-	TGACTA**CCCTAGACCTGACCTTGACAACA**-48 bp-**CCCCACGCCCTTCTAAACCCCTG**AAGGTT
2	1407	36.60%	SNP	T → A, T → G, C → T	TGACTA**CCCa**AGACCTGACCTTGACAACA-48 bp-**CCC**CACGCCC**g**TCTAAACCC**t**TGAAGGTT
3	305	7.93%	SNP	T → G, C → T	TGACTA**CCC**TAGACCTGACCTTGACAACA-48 bp-**CCC**CACGCCC**g**TCTAAACCC**t**TGAAGGTT
4	264	6.87%	SNP	T → A	TGACTA**CCCa**AGACCTGACCTTGACAACA-48 bp-**CCC**CACGCCCTTCTAAACCCCTGAAGGTT
5	90	2.34%	3D	CTG	TGACTA**CCC**TAGAC**---**ACCTTGACAACA-48 bp-**CCC**CACGCCCTTCTAAACCCCTGAAGGTT
6	45	1.17%	SNP	A → G	TGACTA**CCC**TAGACCTGACCTTG**g**CAACA-48 bp-**CCC**CACGCCCTTCTAAACCCCTGAAGGTT
007-ref					
1	1140	29.04%	WT	-	TGACTA**CCCTAGACCTGACCTTGACAACA**-48 bp-**CCCCACGCCCTTCTAAACCCCTG**AAGGTT
2	1696	43.21%	SNP	T → A, T → G, C → T	TGACTA**CCCa**AGACCTGACCTTGACAACA-48 bp-**CCC**CACGCCC**g**TCTAAACCC**t**TGAAGGTT
3	409	10.42%	SNP	T → G, C → T	TGACTA**CCC**TAGACCTGACCTTGACAACA-48 bp-**CCC**CACGCCC**g**TCTAAACCC**t**TGAAGGTT
4	291	7.41%	SNP	T → A	TGACTA**CCCa**AGACCTGACCTTGACAACA-48 bp-**CCC**CACGCCCTTCTAAACCCCTGAAGGTT
5	131	3.34%	SNP	C → T	TGACTA**CCC**TAGACCTGACCTTGACAACA-48 bp-**CCC**CACGCCCTTCTAAACCC**t**TGAAGGTT
6	41	1.04%	SNP	T → G, T → G, C → T	TGACTA**CCCg**AGACCTGACCTTGACAACA-48 bp-**CCC**CACGCCC**g**TCTAAACCC**t**TGAAGGTT
008-ref					
1	1511	39.30%	WT	-	TGACTA**CCCTAGACCTGACCTTGACAACA**-48 bp-**CCCCACGCCCTTCTAAACCCCTG**AAGGTT
2	1587	41.28%	SNP	T → A, T → G, C → T	TGACTA**CCCa**AGACCTGACCTTGACAACA-48 bp-**CCC**CACGCCC**g**TCTAAACCC**t**TGAAGGTT
3	241	6.27%	SNP	T → G, C → T	TGACTA**CCC**TAGACCTGACCTTGACAACA-48 bp-**CCC**CACGCCC**g**TCTAAACCC**t**TGAAGGTT
4	230	5.98%	SNP	T → A	TGACTA**CCCa**AGACCTGACCTTGACAACA-48 bp-**CCC**CACGCCCTTCTAAACCCCTGAAGGTT
5	63	1.64%	SNP	C → T	TGACTA**CCC**TAGACCTGACCTTGACAACA-48 bp-**CCC**CACGCCCTTCTAAACCC**t**TGAAGGTT
6	40	1.04%	SNP	T → G, T → G, C → T	TGACTA**CCCg**AGACCTGACCTTGACAACA-48 bp-**CCC**CACGCCC**g**TCTAAACCC**t**TGAAGGTT
pKSE401-AtU6-PDS					
Sort	Reads number	Ratio	variation type	variation	reads seq
001-ref					TGACT-**PAM**----------**sgRNA1 target**----------48 bp-**PAM**----------**sgRNA2 target**----------AAGGTT
1	1558	40.64%	WT	-	TGACTA**CCCTAGACCTGACCTTGACAACA**-48 bp-**CCCCACGCCCTTCTAAACCCCTG**AAGGTT
2	1482	38.65%	SNP	T → A, T → G, C → T	TGACTA**CCCa**AGACCTGACCTTGACAACA-48 bp-**CCC**CACGCCC**g**TCTAAACCC**t**TGAAGGTT
3	284	7.41%	SNP	T → G, C → T	TGACTA**CCC**TAGACCTGACCTTGACAACA-48 bp-**CCC**CACGCCC**g**TCTAAACCC**t**TGAAGGTT
4	224	5.84%	SNP	T → A	TGACTA**CCCa**AGACCTGACCTTGACAACA-48 bp-**CCC**CACGCCCTTCTAAACCCCTGAAGGTT
5	58	1.51%	SNP	T → G, T → G, C → T	TGACTA**CCCg**AGACCTGACCTTGACAACA-48 bp-**CCC**CACGCCC**g**TCTAAACCC**t**TGAAGGTT
6	41	1.07%	SNP	T → A, T → G	TGACTA**CCCa**AGACCTGACCTTGACAACA-48 bp-**CCC**CACGCCC**g**TCTAAACCCCTGAAGGTT
002-ref					
1	1549	40.33%	WT	-	TGACTA**CCCTAGACCTGACCTTGACAACA**-48 bp-**CCCCACGCCCTTCTAAACCCCTG**AAGGTT
2	1942	38.84%	SNP	T → A, T → G, C → T	TGACTA**CCCa**AGACCTGACCTTGACAACA-48 bp-**CCC**CACGCCC**g**TCTAAACCC**t**TGAAGGTT
3	296	7.70%	SNP	T → G, C → T	TGACTA**CCC**TAGACCTGACCTTGACAACA-48 bp-**CCC**CACGCCC**g**TCTAAACCC**t**TGAAGGTT
4	263	6.85%	SNP	T → A	TGACTA**CCCa**AGACCTGACCTTGACAACA-48 bp-**CCC**CACGCCCTTCTAAACCCCTGAAGGTT
5	47	1.22%	SNP	T → A, T → G	TGACTA**CCCa**AGACCTGACCTTGACAACA-48 bp-**CCC**CACGCCC**g**TCTAAACCCCTGAAGGTT
6	40	1.04%	SNP	T → G, T → G, C → T	TGACTA**CCCg**AGACCTGACCTTGACAACA-48 bp-**CCC**CACGCCC**g**TCTAAACCC**t**TGAAGGTT
003-ref					
1	1427	37.75%	WT	-	TGACTA**CCCTAGACCTGACCTTGACAACA**-48 bp-**CCCCACGCCCTTCTAAACCCCTG**AAGGTT
2	1558	41.22%	SNP	T → A, T → G, C → T	TGACTA**CCCa**AGACCTGACCTTGACAACA-48 bp-**CCC**CACGCCC**g**TCTAAACCC**t**TGAAGGTT
3	277	7.33%	SNP	T → G, C → T	TGACTA**CCC**TAGACCTGACCTTGACAACA-48 bp-**CCC**CACGCCC**g**TCTAAACCC**t**TGAAGGTT
4	258	6.83%	SNP	T → A	TGACTA**CCCa**AGACCTGACCTTGACAACA-48 bp-**CCC**CACGCCCTTCTAAACCCCTGAAGGTT
5	55	1.46%	SNP	T → G, T → G, C → T	TGACTA**CCCg**AGACCTGACCTTGACAACA-48 bp-**CCC**CACGCCC**g**TCTAAACCC**t**TGAAGGTT
6	49	1.30%	SNP	T → A, A → G, T → G, C → T	TGACTA**CCCa**AGACCTGACCTTG**g**CAACA-48 bp-**CCC**CACGCCC**g**TCTAAACCC**t**TGAAGGTT
004-ref					
1	1531	40.47%	WT	-	TGACTA**CCCTAGACCTGACCTTGACAACA**-48 bp-**CCCCACGCCCTTCTAAACCCCTG**AAGGTT
2	1485	39.26%	SNP	T → A, T → G, C → T	TGACTA**CCCa**AGACCTGACCTTGACAACA-48 bp-**CCC**CACGCCC**g**TCTAAACCC**t**TGAAGGTT
3	308	8.14%	SNP	T → G, C → T	TGACTA**CCC**TAGACCTGACCTTGACAACA-48 bp-**CCC**CACGCCC**g**TCTAAACCC**t**TGAAGGTT
4	293	7.75%	SNP	T → A	TGACTA**CCCa**AGACCTGACCTTGACAACA-48 bp-**CCC**CACGCCCTTCTAAACCCCTGAAGGTT
005-ref					
1	1633	43.64%	WT	-	TGACTA**CCCTAGACCTGACCTTGACAACA**-48 bp-**CCCCACGCCCTTCTAAACCCCTG**AAGGTT
2	1422	38.00%	SNP	T → A, T → G, C → T	TGACTA**CCCa**AGACCTGACCTTGACAACA-48 bp-**CCC**CACGCCC**g**TCTAAACCC**t**TGAAGGTT
3	231	6.17%	SNP	T → A	TGACTA**CCCa**AGACCTGACCTTGACAACA-48 bp-**CCC**CACGCCCTTCTAAACCCCTGAAGGTT
4	211	5.64%	SNP	T → G, C → T	TGACTA**CCC**TAGACCTGACCTTGACAACA-48 bp-**CCC**CACGCCC**g**TCTAAACCC**t**TGAAGGTT
5	90	2.41%	1D,SNP	T → A, A, T → G, C → T	TGACTA**CCCa**AGACCTGACCTTGACAACA-48 bp-**CCC**C**-**CGCCC**g**TCTAAACCC**t**TGAAGGTT
006-ref					
1	1389	35.63%	WT	-	TGACTA**CCCTAGACCTGACCTTGACAACA**-48 bp-**CCCCACGCCCTTCTAAACCCCTG**AAGGTT
2	1449	37.18%	SNP	T → A, T → G, C → T	TGACTA**CCCa**AGACCTGACCTTGACAACA-48 bp-**CCC**CACGCCC**g**TCTAAACCC**t**TGAAGGTT
4	360	9.23%	SNP	T → A	TGACTA**CCCa**AGACCTGACCTTGACAACA-48 bp-**CCC**CACGCCCTTCTAAACCCCTGAAGGTT
3	352	9.03%	SNP	T → G, C → T	TGACTA**CCC**TAGACCTGACCTTGACAACA-48 bp-**CCC**CACGCCC**g**TCTAAACCC**t**TGAAGGTT
5	85	2.18%	SNP	T → A, C → T, T → G, C → T, 1D	TGACTA**CCCa**AGACCTGACCTTGACAACA-48 bp-**CCCt**ACGCCC**g**TCTAAACC**-t**TGAAGGTT
007-ref					
1	1508	38.66%	WT	-	TGACTA**CCCTAGACCTGACCTTGACAACA**-48 bp-**CCCCACGCCCTTCTAAACCCCTG**AAGGTT
2	1494	38.31%	SNP	T → A, T → G, C → T	TGACTA**CCCa**AGACCTGACCTTGACAACA-48 bp-**CCC**CACGCCC**g**TCTAAACCC**t**TGAAGGTT
3	300	7.69%	SNP	T → A	TGACTA**CCCa**AGACCTGACCTTGACAACA-48 bp-**CCC**CACGCCCTTCTAAACCCCTGAAGGTT
4	228	5.84%	SNP	T → G, C → T	TGACTA**CCC**TAGACCTGACCTTGACAACA-48 bp-**CCC**CACGCCC**g**TCTAAACCC**t**TGAAGGTT
5	64	1.64%	SNP	C → T	TGACTA**CCC**TAGACCTGACCTTGACAACA-48 bp-**CCC**CACGCCCTTCTAAACCC**t**TGAAGGTT
6	61	1.56%	SNP	T → A, T → G, T → C, C → T	TGACTA**CCCa**AGACCTGACCTTGACAACA-48 bp-**CCC**CACGCCC**gc**CTAAACCC**t**TGAAGGTT
7	48	1.23%	SNP	T → G, T → G, C → T	TGACTA**CCCg**AGACCTGACCTTGACAACA-48 bp-**CCC**CACGCCC**g**TCTAAACCC**t**TGAAGGTT
8	40	1.03%	1I,1D	G, G	TGACTA**CCC**TAGACCT**-**ACCTTGACAACA-48 bp-**CCC**CACGCCCTTCTAAACCCCT**G**GAaGTT
008-ref					
1	1580	41.75%	WT	-	TGACTA**CCCTAGACCTGACCTTGACAACA**-48 bp-**CCCCACGCCCTTCTAAACCCCTG**AAGGTT
2	1321	34.91%	SNP	T → A, T → G, C → T	TGACTA**CCCa**AGACCTGACCTTGACAACA-48 bp-**CCC**CACGCCC**g**TCTAAACCC**t**TGAAGGTT
3	349	9.22%	SNP	T → G, C → T	TGACTA**CCC**TAGACCTGACCTTGACAACA-48 bp-**CCC**CACGCCC**g**TCTAAACCC**t**TGAAGGTT
4	235	6.21%	SNP	T → A	TGACTA**CCCa**AGACCTGACCTTGACAACA-48 bp-**CCC**CACGCCCTTCTAAACCCCTGAAGGTT
5	68	1.80%	3D	CTG	TGACTA**CCC**TAGAC**---**ACCTTGACAACA-48 bp-**CCC**CACGCCCTTCTAAACCCCTGAAGGTT
6	43	1.14%	SNP	T → G, T → G, C → T	TGACTA**CCCg**AGACCTGACCTTGACAACA-48 bp-**CCC**CACGCCC**g**TCTAAACCC**t**TGAAGGTT
7	40	1.06%	SNP	T → A, T → G	TGACTA**CCCa**AGACCTGACCTTGACAACA-48 bp-**CCC**CACGCCC**g**TCTAAACCCCTGAAGGTT

Column 1 (Sort): Sample names and mutation types derived from sequencing analysis. Each sample name occupies a separate row, structured as: Sample Name—Matched Reference Sequence Name. Entries consisting solely of numbers below indicate mutation types. Column 2 (Reads number): the number of mutated sequences obtained from high-throughput sequencing, equivalent to the number of T-vector single clones picked. Column 3 (Ratio): the proportion of this mutation type within the sample. Column 4 (Variation type): mutation types within 100 bp of the primer, where 1D represents a single base deletion, 1I indicates a single base insertion, and SNP stands for a single nucleotide polymorphism. Column 5 (Variation): information on the mutated nucleotide within 100 bp of the primer. Column 6 (Reads seq): the sequencing sequence, with mutated positions indicated by lowercase letters.
